# Association of non-nutritive sweetener consumption with food craving and body image among university students: A cross-sectional study

**DOI:** 10.1371/journal.pone.0335838

**Published:** 2025-12-04

**Authors:** Anfal AL-Dalaeen, Hebah Hamid, Tasneem Abu Hasan

**Affiliations:** Department of Clinical Nutrition and Dietetics, Faculty of Allied Medical Sciences, Applied Science Private University, Amman, Jordan; University of Petra (UOP), JORDAN

## Abstract

Non-nutritive sweeteners (NNS) are widely used for weight control and dietary regulation by university students, however, their psychological associations remain unclear. The aim of this study to investigate the associations between NNS consumption and food craving, and body dissatisfaction. A cross-sectional analysis was conducted among 300 students from Applied Science Private University (ASU) in Amman, Jordan. Participants completed a validated online questionnaire including demographic data, NNS consumption, food craving using Food Craving Questionnaire-Trait Rediced (FCQ-Tr), and perceptions of Sihhouette Ratine Scale Dissatisfcation index (SRS-D) body image using. To evaluate the associations between NNS exposure and psychological outcomes, age, sex, body mass index (BMI), dieting status, smoking behavior, were adjusted using multivariable linear and ordinal regression models. The NNS consumption was 83.3% among university students, with 30.0% indicating daily use of NNS. Increased frequency of NNS intake was significantly associated with higher FCQ-T-r scores (β = 0.21, 95% CI [0.08–0.34], p = 0.002) and elevated SRS-D scores (β = 0.17, 95% CI [0.05–0.29], p = 0.006). Also (74.3%) reported no mood change after NNS intake, and 21.0% reported mood enhancement. These findings found that frequent NNS = intake is associated with heightened food craving and body dissatisfaction among students, independent of body weight, age, smoking or academic faculty.

## 1. Introduction

Non-nutritive sweeteners (NNS), is known as artificial sweeteners or low-calorie sweeteners, which are widely used as sugar substitutes because they provide sweetness without adding significant calories [1]. Their use has increased considerably over the past decades, particularly among young adults and university students, who are concerned with weight management and metabolic health [1,2]. NNS are found in a variety of products, including beverages, desserts, and processed foods [3]. However, the effects of NNS on dietary behaviors and psychological parameter remain unclear, with studies in consistence result.

One of concern area is the influence of NNS on food cravings. Food craving, is intense desire to consume foods high in sugar or fat, has been linked to overeating, weight gain, and poor dietary quality [4,5]. Some studies suggest NNS consumption may paradoxically increase cravings by altering reward system or disrupting appetite regulation hormones [6,7], while others indicate they reduce caloric intake by substituting for sugar [2,4]. Understanding this relationship is particularly relevant for university students, as irregular eating habits, stress levels, and lifestyle changes may increase susceptibility to food cravings and dysregulated eating behavior and dietary choices.

Body image, which defined as an individuals perception, attitudes, and satisfaction with one’s physical appearance [8]. University students are at a heightened risk of body dissatisfaction due to societal pressures, media influence, and peer comparison [9]. Body image concerns can strongly influence dietary behaviors, including the use of NNS, for weight control [10]. However, whether higher body dissatisfaction is directly related to NNS use remains unclear.

Most studies focus on metabolic outcomes, weight management, [3,11].However, evidence exploring food craving and body-image domains remains sparse, particularly in non-Western settings. To date, no studies in Jordan have examined how NNS consumption may relate to food cravings or perceived body image among university students, despite cultural and dietary trends suggesting rising NNS use.

Therefore, this study aimed to investigate the associations between NNS consumption, food craving, and body image dissatisfaction among university students in Amman, Jordan. Specifically, we examined whether the frequency and form of NNS use were associated with higher craving and whether these associations persisted after controlling for confounding factors such as age, sex, BMI category, dieting status, smoking, and academic faculty.

## 2. Materials and methods

### 2.1. Study design and sample size

This cross-sectional study was conducted among university students to examine the association between NNS consumption, food cravings, and body image dissatisfaction. A total of 300 students aged 18 years and above were recruited from Applied Science Private University (ASU) between May and July 2025.

A priori sample-size estimation targeted detection of small-to-moderate effects (r ≈ 0.20 or β ≈ 0.15) at α = 0.05 and 80% power, requiring at least 194 participants. Post-hoc achieved power (β = 0.20) = 0.84 was also reported for transparency. Inclusion criteria included being enrolled at ASU, being 18 years or older, and providing complete questionnaire responses. Exclusion criteria were incomplete data or self-reported chronic disease affecting appetite or weight regulation.

Ethics approval was obtained from the Research Ethics Committee, Faculty of Allied Medical Sciences, Applied Science Private University (Approval No. AMS-11–2025).

### 2.2. Measures

#### 2.2.1. Sociodemographic and lifestyle characteristics.

Participants reported Age, gender, academic faculty (health vs non-health), body weight, height, smoking status, and medication use were reported by participants. BMI was calculated as weight (kg)/height^2^ (m^2^) and categorized using WHO criteria [12].

#### 2.2.2. Non-nutritive sweeteners (NNS) consumption.

The NNS consumption was assessed using a questionnaire adapted from Webb et al. (2021) [1], which was including frequency, type of NNS, and perceived mood effects. The scale demonstrated acceptable reliability in this sample (Cronbach’s α = 0.73, 95% CI [0.68–0.78]), and face validity was confirmed through expert review by faculty members in the Department of Clinical Nutrition, Faculty of Allied Medical Sciences. Details of the Arabic version, scoring instructions, and translation/back-translation procedures are detailed in the Supplementary Material S1 File.

#### 2.2.3. Body image.

Body image perception was assessed using validated the Silhouette Rating Scale test [2] (SRS; Lombardo et al., 2022), a validated pictorial tool consisting of nine gender-specific silhouettes ranging from very thin (score = 1) to very large (score = 9). Participants selected the silhouette representing their current body size (SRS-C) and ideal body size (SRS-I). Body dissatisfaction (SRS-D) was calculated as SRS-I – SRS-C, with negative scores indicating a desire to be thinner and positive scores indicating a desire to be larger.

#### 2.2.4. Food cravings.

Food cravings were assessed using the Food Craving Questionnaire–Trait, Reduced (FCQ-T-r), developed by Meule et al. (2014) [3], a validated 15-item scale measuring the frequency and intensity of trait-level food cravings. Each item is rated on a 5-point Likert scale (1 = never to 5 = always), with total scores ranging from 15–75. Higher scores indicate higher cravings. The internal consistency in this sample was α = 0.83 (95% CI [0.79–0.87]), including its Arabic translation. Full scoring instructions and translation procedures are provided in the Supplementary Material S1 File.

### 2.3. Statistical analysis

Analyses were performed using IBM SPSS Statistics version 27 (IBM Corp., USA). Descriptive statistics (mean ± SD, frequencies, and percentages). Multivariable regression test was used to examine associations between NNS exposure and psychological outcomes. Linear regression measured the continuous outcomes (FCQ-T-r and DEQ dissatisfaction), while ordinal logistic regression examined SRS-D. Predictors included NNS frequency and form, adjusted for age, sex, BMI category, dieting status, smoking, medication use, and faculty.

Model diagnostics verified residual normality, homoscedasticity, and multicollinearity (variance-inflation factor < 5). Results were expressed as standardized regression coefficients (β) with 95% confidence intervals (CIs). Multiple comparisons were controlled using the false-discovery-rate (FDR) method, and statistical significance was set at p < 0.05. Missing data (< 2%) were managed through listwise deletion after confirmation of randomness (Little’s MCAR test p > 0.10).

## 3. Results

### 3.1. Participant characteristics

A total of 300 students participated in the study. Most were aged 18–24 years (81.5%), followed by 25–29 years (13.4%) and 30–40 years (5.1%). Females constituted 56.6% of the sample, and students were evenly distributed between health (50.3%) and non-health (49.7%) faculties. The majority were non-smokers (66.6%), not currently dieting (66.7%), and classified as normal weight (52.0%), with 29.8% overweight and 11.6% obese (Table 1).

**Table 1:** Sociodemographic and lifestyle characteristics of participants (*n *= 300).

Variable	Category	Frequency (*n*)	Percentage (%)
**Age**	18–24 years	245	81.5
	25–29 years	40	13.4
	30–40 years	15	5.1
**Gender**	Male	130	43.4
	Female	170	56.6
**Faculty**	Health	151	50.3
	Non-health	149	49.7
**Smoking status**	Yes	100	33.4
	No	200	66.6
**Currently on a weight-loss diet**	Yes	100	33.3
	No	200	66.7
**Body Mass Index categories**	Underweight	20	6.6
	Normal weight	156	52.0
	Overweight	89	29.8
	Obese	35	11.6

Data are presented as unadjusted frequencies and percentages, calculated from the total sample (*n* = 300) unless otherwise stated.

### 3.2. Non-nutritive sweetener consumption patterns

Table 2 summarizes NNS usage patterns. More than half of participants (56.7%) had ever used NNS, and 83.3% reported current consumption of NNS containing foods or beverages. Pill or powder forms were used by 30.0% of students. Regarding frequency, 33.3% reported daily use, 40.0% weekly, and 26.7% rarely. Most participants (73.3%) were uncertain about differences between sweetener types, whereas 21.7% believed that no type was superior

**Table 2:** Usage patterns of non-nutritive sweeteners among participants (*n* = 300).

Variable	Category	Frequency (*n*)	Percentage (%)
**Ever used NNS**	Yes	170	56.7
	No	130	43.3
**Current consumption of NNS foods/beverages**	Yes	250	83.3
	No	50	16.7
**Consumption of NNS (pill/powder)**	Yes	90	33.0
	No	210	70.0
**Frequency of NNS use**	Less than once a week	80	26.7
	Daily	100	33.3
	Once a week	120	40.0
**Perception of one type being better**	NNS Uncertain/ Don’t know	220	73.3
	No type is better	65	21.7
	Stevia is best	4	1.3
	Aspartame is best	3	1.0
	Sucralose is best	6	2.0
	Ace-K is best	2	0.7

Data are presented as unadjusted frequencies and percentages, calculated from the total sample (*n* = 300). NNS; Non-nutritive Sweeteners.

### 3.3. Food-craving–related behaviors following NNS consumption

Table 3 presents behaviors and subjective responses after NNS intake. The majority (74.3%) reported no change in mood, 21.0% reported a positive mood change, and 4.7% reported a negative change. Approximately 74.6% indicated that they could control the type and quantity of food they consumed, while 25.4% reported reduced control. A quarter of participants (25.0%) experienced an increased desire for sweets or fast food, whereas 75.0% did not. Based on craving-intensity categorization, 30.0% experienced low, 9.1% moderate, and 10.0% high levels of food craving.

**Table 3:** Food Cravings and Related Behaviors Following Non-nutritive Sweeteners Consumption (*n *= 300).

Variable	Category	Frequency (*n*)	Percentage (%)
**Mood change after NNS**	None	223	74.3
	Negative	14	4.7
	Positive	63	21.0
**Control over food type/quantity**	Yes	224	74.6
	No	76	25.4
**Increased desire for sweets/fast food**	No	225	75.0
	Yes	75	25.0
	No change in desire	52	17.6
	Neutral response	100	33.3
**Desire for food (craving level)**	Low craving	90	30.0
	Moderate craving	28	9.1
	High craving	30	10.0

Data are presented as unadjusted frequencies and percentages among current NNS consumers (n = 300) NNS; Non-nutritive Sweeteners.

### 3.4. Mean scores of food-craving and body-image variables

Table 4 summarizes mean scores and effect-size correlations for food craving and body image variables. The mean food-craving score was 30.5 ± 1.8, indicating moderate craving intensity. The mean current body-size rating (SRS-C) was 5.3 ± 1.8, while the ideal body-size rating (SRS-I) was 5.3 ± 1.0. The resulting body-dissatisfaction score (SRS-D) was –1.35 ± 1.50, reflecting a general desire to be thinner. The DEQ-based dissatisfaction score averaged 8.9 ± 6.1 (p < 0.001). Effect-size correlations demonstrated that craving scores were positively correlated with current body size (r = 0.249, 95% CI [0.135, 0.355]) and ideal body size (r = 0.281, 95% CI [0.170, 0.384]), whereas DEQ dissatisfaction was negatively correlated with SRS-D (r = –0.344, 95% CI [–0.445, –0.235]). Frequency of NNS use showed a weak but significant correlation with ideal body size (r = 0.158, 95% CI [0.032, 0.278]).

**Table 4:** Mean Scores of Body Image and Food Craving Variables (*n *= 300).

Variable	Mean± SD	p-value	Effect Size (r)	95% CI for r
Craving Score	30.5 ± 1.8	-	-	_
Current body size (SRS-C)	5.3 ± 1.8		0.249	[0.135, 0.355]
Ideal body size (SRS-I)	5.3 ± 1.0		0.281	[0.170, 0.384]
Dissatisfaction Score (SRS-D)	−1.35 ± 1.5		–0.344	[–0.445, –0.235]
DEQ dissatisfaction	8. ± 6.1	0.001		

Data are presented as unadjusted means ± standard deviation (SD). SRS-C; Silhouette Rating Scale – Current Body Size; SRS-I; Silhouette Rating Scale – Ideal Body Size; SRS-D; Body Dissatisfaction Score (SRS-I – SRS-C); DEQ; Dietary Evaluation Questionnaire; FCQ-T-r; Food Craving Questionnaire–Trait Reduced; NNS; Non-nutritive Sweeteners. Effect sizes (r) are reported with 95% confidence intervals (CI). Primary outcomes: FCQ-T-r and SRS-D; secondary outcome: DEQ dissatisfaction.

### 3.5. Correlational and multivariable analyses

As shown in Table 5, Pearson correlation analyses indicated that food-craving scores were positively correlated with both current body-size perception (SRS-C; r = 0.249, p < 0.01, 95% CI [0.135–0.355]) and ideal body size (SRS-I; r = 0.281, p < 0.01, 95% CI [0.170–0.384]). Body-dissatisfaction scores (SRS-D) were negatively correlated with dietary-evaluation (DEQ) dissatisfaction (r = –0.344, p < 0.01, 95% CI [–0.445– –0.235]), suggesting that greater perceived discrepancy between current and ideal body size corresponded to higher overall dissatisfaction. A weak but statistically significant correlation emerged between frequency of NNS use and ideal body size (r = 0.158, p < 0.05, 95% CI [0.032–0.278]), indicating that participants who consumed NNS more frequently tended to idealize a smaller or leaner body shape.

**Table 5:** Pearson Correlations Between Food Cravings, Body Dissatisfaction, and NNS Use.

Variable	r-value	p-value	95% CI for r
**Craving score × SRS-C**	0.249	<0.01	[0.135, 0.355]
**Craving score × SRS-I**	0.281	<0.01	[0.170, 0.384]
**Body dissatisfaction (SRS-D) × DEQ dissatisfaction**	−0.344	<0.01	[–0.445, –0.235]
**Frequency of NNS use × SRS-I**	0.158	<0.05	[0.032, 0.278]

Values represent unadjusted Pearson correlation coefficients (r) with 95% confidence intervals (CI). p values are FDR-adjusted for multiple testing. Analyses not pre-specified a priori are considered exploratory. SRS-C; Silhouette Rating Scale – Current Body Size; SRS-I; Silhouette Rating Scale – Ideal Body Size; SRS-D; Body Dissatisfaction Score (SRS-I – SRS-C); DEQ; Dietary Evaluation Questionnaire; NNS; Non-nutritive Sweeteners; FDR; False Discovery Rat

### 3.6. Multivariable regression models

As shown in Table 6, multivariable regression analyses adjusted for age, sex, BMI category, dieting status, smoking, medication use, and academic faculty confirmed that frequency of NNS use remained an independent predictor of all three psychological outcomes. Specifically, higher NNS frequency was associated with greater food-craving intensity (FCQ-T-r: β = 0.21, 95% CI [0.08–0.34], p = 0.002; R^2^ = 0.185), higher body-image dissatisfaction (SRS-D: β = 0.17, 95% CI [0.05–0.29], p = 0.006; Nagelkerke R^2^ = 0.14), and greater dietary dissatisfaction (DEQ: β = 0.19, 95% CI [0.06–0.32], p = 0.004; R^2^ = 0.162).

**Table 6:** Multivariable Regression Models Examining Associations Between NNS Consumption and Psychological Outcomes (*n* = 300).

Outcome Variable	Predictor Frequency of NNS use	Adjusted β (95% CI)	p-value (FDR-adjusted)	R²
**FCQ-T-r (Food Craving Score)**	Frequency of NNS use	0.21[0.08–0.34]	**0.002**	**0.185**
**SRS-D (Body Dissatisfaction†**	Frequency of NNS use	0.17[0.05–0.29]	**0.006**	**0.14**
**DEQ (Dietary Evaluation Questionnaire) Dissatisfaction**	Frequency of NNS use	0.19[0.06–0.32]	**0.004**	**0.162**

Values represent adjusted standardized regression coefficients (β) with 95% confidence intervals (CI). All models were adjusted for age, sex, BMI category, dieting status, smoking status, medication use, and academic faculty. p values are FDR-adjusted for multiple testing. FCQ-T-r; Food Craving Questionnaire–Trait Reduced; SRS-D; Silhouette Rating Scale – Dissatisfaction score; DEQ; Dietary Evaluation Questionnaire; NNS; Non-nutritive Sweeteners; FDR; False Discovery Rate. Primary outcomes: FCQ-T-r and SRS-D; secondary outcome: DEQ dissatisfaction.

## 4. Discussion

This study examined the relationship between NNS consumption, food craving, and body image among university students. To our knowledge, it is the first study to explore the psychological and behavioral correlations of NNS use in this population. The results demonstrated that NNS consumption was highly prevalent—reported by more than four out of five participants—and that greater frequency of NNS use was associated with higher craving intensity, greater body-image dissatisfaction, and increased dietary dissatisfaction, even after adjusting for demographic and lifestyle factors.

The results demonstrate a high percentage of NNS intake, reported by 83.3% of participants, with 30% using NNS daily and a similar proportion consuming them in pill or powder form. This result is consistent with global trends, showing that young adults increasingly substitute sugar with NNS to manage body weight or decrease calorie intake [1,2]. However, frequent NNS consumption has raised concerns about potential effects on appetite regulation, metabolic responses, and eating behaviors [11]. Despite most participants (74.3%) reporting no mood change after NNS consumption and 21% reporting a positive shift, the adjusted regression models revealed that NNS frequency independently predicted higher food-craving intensity, greater body-image dissatisfaction, and higher dietary dissatisfaction, even after controlling for BMI, dieting status.

The magnitude of these associations (β ≈ 0.17–0.21; R^2^ = 0.14–0.19) indicates that NNS consumption frequency meaningfully contributes to psychological variability, though other factors such as dieting motivation, stress, and exposure to food cues likely moderate these effects. These results align with behavioral-nutrition studies suggesting that NNS exposure may alter appetite control, enhance reward sensitivity, and disrupt satiety signaling [13,14]. Similar findings by Yunker et al. (2020) and Haroun et al. (2025) support the notion that habitual NNS intake may be linked to stronger food-craving tendencies and heightened body-image concern among young adults [2,3].

The lack of associations of the NNS form (pill/powder versus beverage) implies that usage patterns rather than NNS type may drive these relationships. A Study indicates that NNS activate sweet taste receptors in the mouth and gut, stimulating T1R2/T1R3 receptors without actual caloric intake [11]. This activation triggers neural responses associated with caloric intake, creating an expectation of energy that is not met due to the absence of real calories [3,13]. However, these effects appear highly individual and may be moderated by psychological factors such as dietary restraint and habitual sweetener use [12]. In the current study, the lack of association between NNS consumption and mood or craving intensity among university students suggests that such mechanisms are not universal and may be buffered by behavioral control, dietary restraint, or habitual sweetener exposure.

Correlation analyses additionally indicated a larger perceived or ideal body size was associated with higher craving scores (SRS-I: r = 0.281, p < 0.01), while greater discrepancy between current and ideal body size corresponded to higher dissatisfaction (r = –0.344, p < 0.01). Moreover, the positive association between NNS frequency and ideal body size (r = 0.158, p < 0.05) suggests that individuals striving for thinner body ideals may rely on NNS as a weight-control strategy, attempting to reduce energy intake while maintaining NNS in their diet. Although direct evidence linking NNS use to body dissatisfaction is limited, these findings are plausible given the social pressures and aesthetic ideals influencing eating behaviors among university populations [11,15]. Potential confounding factors, including diet quality, physical activity, and stress levels, may influence the observed associations between body image, food cravings, and NNS consumption and should be considered in future research to better undertanding of causal effects. From a practical perspective, awareness programs targeting university students could raise understanding of the potential psychological and behavioral impacts of NNS use, thereby supporting healthier dietary choices and encouraging a more positive body image.

This study has several limitations. First, the cross-sectional design limits causal inference and prevents establishing temporal direction between NNS consumption and body image. Second, the sample was collected from a single university in Amman, which may limit generalizability to other populations. Third, the reliance on self-reported data introduces potential recall and social-desirability bias. Fourth, although validated tools were used, constructs such as craving and body image are inherently subjective and may not fully capture cultural or clinical nuances. Fifth, despite adjustment for multiple covariates, residual confounding by unmeasured variables such as stress, dietary restraint, or physical activity cannot be excluded. Finally, multiple comparisons increase the risk of type I error, though false-discovery-rate (FDR) correction was applied to reduce this bias.

## 5. Conclusion

Frequent use of non-nutritive sweeteners (NNS) was common among university students and was independently associated with higher food-craving intensity, greater body-image dissatisfaction, and increased dietary dissatisfaction. These associations persisted after adjustment for demographic and lifestyle variables, suggesting that habitual NNS intake may influence appetite regulation and self-perception of body image. Although causality cannot be determined due to the cross-sectional design, the findings highlight the psychological dimension of NNS consumption. Future longitudinal research should clarify causal pathways and evaluate whether reducing NNS consumption can improve craving control, dietary satisfaction, and overall psychological well-being among university students.

## Supporting information

S1 File Supplementary_Material_Questionnaires. (DOCX)
