# Supplementary material for: Association of non-nutritive sweetener consumption with food craving and body image among university students: A cross-sectional study
Source: PLoS One. 2025 Dec 4;20(12):e0335838. doi: 10.1371/journal.pone.0335838 (PMC12677766; doi:10.1371/journal.pone.0335838)
Supplement: S1 File — (DOCX) [file pone.0335838.s001.docx]

# Supplementary Material

## ****Section A. Non-nutritive Sweetener (NNS) Consumption Questionnaire****

**Instrument Source and Adaptation** This questionnaire was adapted from Webb et al. (2021) to assess NNS consumption patterns among university students. Minor contextual modifications were made to reflect local dietary habits and terminology. Permission for use and adaptation was obtained where required.

**Translation and Validation** Items were translated into Arabic using a forward–backward translation method. Two bilingual experts independently translated the items into Arabic, followed by reconciliation and back-translation into English by a third expert. Discrepancies were resolved through consensus. Face validity was confirmed by faculty members in Clinical Nutrition. Internal consistency in the present sample was acceptable (Cronbach’s α = 0.73; 95% CI [0.68–0.77]).

**English Items**

1. Have you ever consumed non-nutritive sweeteners (NNS)? (Yes / No)
2. Do you currently consume foods or beverages containing NNS? (Yes / No)
3. Do you consume NNS in pill or powder form? (Yes / No)
4. How often do you consume NNS? (Rarely / Once a week / Daily)
5. Do you perceive one type of NNS as better than others? (Specify: None / Stevia / Aspartame / Sucralose / Ace-K / Other)
6. Do you experience mood changes after consuming NNS? (Positive / Negative / None)
7. Do you prefer NNS over sugar in beverages (e.g., coffee, tea, soda)? (Yes / No / Depends on the beverage)

**Arabic Version**

| \| رقم السؤال \| \| السؤال باللغة العربية \| \| --- \| --- \| --- \| \| 1 \| هل سبق لك أن استهلكت مُحليات غير غذائية؟ (نعم / لا) \| \| 2 \| هل تستهلك حاليًا أطعمة أو مشروبات تحتوي على مُحليات غير غذائية؟ (نعم / لا) \| \| 3 \| هل تستهلك المُحليات غير الغذائية على شكل أقراص أو مسحوق؟ (نعم / لا) \| \| 4 \| كم مرة تستهلك المُحليات غير الغذائية؟ (نادرًا / مرة في الأسبوع / يوميًا) \| \| 5 \| هل تعتقد أن نوعًا معينًا من المُحليات أفضل من الأنواع الأخرى؟ (حدد: لا يوجد / ستيفيا / أسبارتام / سوكرالوز / أسيسولفام-ك / أخرى) \| \| 6 \| هل تشعر بتغير في المزاج بعد استهلاك المُحليات غير الغذائية؟ (إيجابي / سلبي / لا يوجد) \| \| 7 \| هل تفضل استخدام المُحليات غير الغذائية بدلًا من السكر في المشروبات (مثل القهوة، الشاي، الصودا)؟ (نعم / لا / حسب نوع المشروب) \| |
| --- | --- | --- | --- | --- | --- | --- | --- | --- | --- | --- | --- | --- | --- | --- | --- | --- | --- |

## ****Section B. Food Craving Questionnaire–Trait, Reduced (FCQ-T-r)****

**Instrument Source** The FCQ-T-r was adapted from Meule et al. (2014), a 15-item scale measuring trait-level food cravings.

**Translation and Reliability.** The Arabic version was developed using forward–backward translation and reviewed by clinical psychologists and nutrition faculty. Internal consistency was excellent (Cronbach’s α = 0.83; 95% CI [0.79–0.86]).

**Scoring Instructions**

- Each item rated on a 5-point Likert scale (1 = never to 5 = always)
- **Total score** = Sum of all 15 items (Range: 15–75)
- Higher scores indicate stronger and more persistent food cravings

**استبيان الرغبة الشديدة في تناول الطعام (FCQ-T-r) النسخة العربية**

يرجى تحديد مدى تكرار كل من العبارات التالية بالنسبة لك، باستخدام المقياس التالي:

| الدرجة | التفسير |
| --- | --- |
| 1 | أبدًا |
| 2 | نادرًا |
| 3 | أحيانًا |
| 4 | غالبًا |
| 5 | دائمًا |

| رقم | العبارة |
| --- | --- |
| 1 | أفكر كثيرًا في الطعام. |
| 2 | أشعر برغبة قوية في تناول أطعمة معينة. |
| 3 | أجد صعوبة في مقاومة الرغبة في تناول الطعام. |
| 4 | أتناول الطعام حتى عندما لا أكون جائعًا. |
| 5 | أشعر بالحاجة لتناول الطعام عندما أكون متوترًا أو قلقًا. |
| 6 | أجد نفسي أبحث عن الطعام دون تخطيط مسبق. |
| 7 | أتناول الطعام كوسيلة للشعور بالراحة. |
| 8 | أشعر أنني لا أستطيع التوقف عن التفكير في الطعام. |
| 9 | أتناول الطعام بسرعة عندما أشعر برغبة شديدة. |
| 10 | أتناول الطعام حتى عندما أعلم أنه غير صحي. |
| 11 | أتناول الطعام في أوقات غير معتادة (مثل منتصف الليل). |
| 12 | أشعر أنني أحتاج إلى تناول الطعام فورًا. |
| 13 | أتناول الطعام عندما أشعر بالملل. |
| 14 | أتناول الطعام عندما أكون حزينًا أو محبطًا. |
| 15 | أتناول الطعام عندما أكون سعيدًا أو أحتفل. |

**تعليمات احتساب الدرجات:**

- الدرجة الكلية = مجموع درجات البنود الـ15 (النطاق: 15–75).
- كلما ارتفعت الدرجة، زادت شدة وتكرار الرغبة في تناول الطعام كصفة شخصية.
